# Clinical Predictive Score for Identifying Metabolic Dysfunction-Associated Steatotic Liver Disease in Individuals with Prediabetes Using Transient Elastography

**DOI:** 10.3390/jcm12247617

**Published:** 2023-12-11

**Authors:** Nutthachoke Mahachai, Chaiwat Washirasaksiri, Pinyapat Ariyakunaphan, Chayanis Kositamongkol, Tullaya Sitasuwan, Rungsima Tinmanee, Chonticha Auesomwang, Naruemit Sayabovorn, Thanet Chaisathaphol, Pochamana Phisalprapa, Phunchai Charatcharoenwitthaya, Weerachai Srivanichakorn

**Affiliations:** 1 Department of Medicine, Faculty of Medicine Siriraj Hospital, Mahidol University, Bangkok 10700, Thailand; nutthachoke.m@cpird.in.th; 2 Division of Ambulatory Medicine, Department of Medicine, Faculty of Medicine Siriraj Hospital, Mahidol University, Bangkok 10700, Thailand; chaiwat.was@mahidol.ac.th (C.W.); pochamana.phi@mahidol.ac.th (P.P.); 3 Division of Gastroenterology, Department of Medicine, Faculty of Medicine Siriraj Hospital, Mahidol University, Bangkok 10700, Thailand

**Keywords:** metabolic dysfunction-associated steatotic liver disease, prediabetic state, screening and diagnostic method, Thais, transient elastography

## Abstract

Scoring systems for metabolic dysfunction-associated steatotic liver disease (MASLD) in individuals with prediabetes have not been extensively explored. This study aimed to investigate the prevalence of MASLD and to develop predictive tools for its detection in high cardiometabolic people with prediabetes. A cross-sectional study was conducted using baseline data from the prediabetes cohort. All participants underwent transient elastography to assess liver stiffness. MASLD was defined using a controlled attenuation parameter value > 275 dB/m and/or a liver stiffness measurement ≥ 7.0 kPa. Cases with secondary causes of hepatic steatosis were excluded. Out of 400 participants, 375 were included. The observed prevalence of MASLD in individuals with prediabetes was 35.7%. The most effective predictive model included FPG ≥ 110 mg/dL; HbA1c ≥ 6.0%; sex-specific cutoffs for HDL; ALT ≥ 30 IU/L; and BMI levels. This model demonstrated good predictive performance with an AUC of 0.80 (95% CI 0.73–0.86). At a cutoff value of 4.5, the sensitivity was 70.7%, the specificity was 72.3%, the PPV was 58.8%, and the NPV was 81.5%. Our predictive model is practical, easy to use, and relies on common parameters. The scoring system should aid clinicians in determining when further investigations of MASLD are warranted among individuals with prediabetes, especially in settings with limited resources.

## 1. Introduction

Prediabetes is a medical condition characterized by hyperglycemia levels above normal but below the threshold for diabetes. It poses a high risk for the development of diabetes, atherosclerotic cardiovascular disease, and associated metabolic complications [1]. Metabolic dysfunction-associated steatotic liver disease (MASLD) is a leading cause of chronic liver disease and is commonly associated with insulin resistance, metabolic syndrome, prediabetes, and type 2 diabetes [2]. Prediabetes and MASLD share similar risk factors, including being overweight, obese, or having central obesity. These factors contribute to systemic insulin resistance and increased levels of circulating free fatty acids, which are subsequently stored in the liver, resulting in MASLD. This hepatic fat accumulation promotes hepatic insulin resistance, activates inflammatory pathways, increases oxidative stress, and leads to hepatic fibrosis [3].

Previous studies have shown that individuals with both prediabetes and MASLD have a higher risk of developing type 2 diabetes than those with prediabetes alone [4]. Additionally, MASLD is associated with an increased risk of cirrhosis, cardiovascular disease, and cancer [5,6]. Typically, MASLD patients remain asymptomatic until severe liver disease manifests. Therefore, early detection of MASLD is crucial to prevent disease progression and associated complications. While liver biopsy is the gold standard for diagnosing metabolic dysfunction-associated steatohepatitis (MASH), its invasiveness and potential complications limit its widespread use [7]. Noninvasive approaches, such as transient elastography, have been developed and have shown good sensitivity in detecting MASLD and significant fibrosis [8,9,10,11]. However, the exact prevalence of MASLD is still ambiguous and varies depending on the screening methods used and the nature of the populations studied.

The prevalence of MASLD in individuals with prediabetes, particularly in the Asian population, has not been extensively studied. Therefore, this study sought to investigate the prevalence of MASLD, identify predictive risk factors, and develop a simple clinical predictive score for detecting MASLD in Thai individuals with prediabetes. Furthermore, we aimed to compare our predictive tool with previously published tools designed for predicting MASLD.

## 2. Materials and Methods

A cross-sectional analysis was conducted using the baseline data from a cohort of individuals with high cardiometabolic risk and prediabetes who attended the outpatient clinic of the Faculty of Medicine Siriraj Hospital between 2019 and 2022. Typically, attendees of this clinic are at high risk for diabetes or cardiovascular diseases, or they already have conditions such as hypertension, obesity, or multiple metabolic risk factors. The clinic provides education, prevention strategies, and appropriate medications such as blood pressure-lowering agents and cholesterol-lowering agents.

Participants included in the study were aged between 18 and 80 years and had experienced glucose levels within the prediabetes range on at least two occasions. Prediabetes was defined as a hemoglobin A1c (HbA1c) level of 39 to less than 48 mmol/mol (5.7% to less than 6.5%) and/or a fasting plasma glucose (FPG) level of 100 to less than 126 mg/dL [1]. All participants underwent transient elastography. Additionally, participants were required to have no previous diagnosis of diabetes, not be using hypoglycemic agents, and not be pregnant or breastfeeding.

Individuals with any secondary cause of hepatic steatosis were excluded, including those with a history of excessive alcohol intake (more than 30 g/day for men and 20 g/day for women), chronic viral hepatitis (hepatitis B or C), chronic liver disease, or drug-induced hepatitis. After applying these exclusion criteria, 375 participants were recruited for the present study.

The study protocol was approved by the Siriraj Institutional Review Board (certificate of approval [COA] number SI 495/2019 and COA number SI 447/2023).

### 2.1. Procedures and Measurements

All recruited individuals with prediabetes underwent various measurements and assessments. These included blood pressure and anthropometric measurements (such as height, weight, and waist circumference) which were conducted prior to performing the transient elastography. Transient elastography using the FibroScan Compact 530 (probe M+; Echosens, Paris, France) was also performed by one trained technician. Furthermore, the study obtained details of common predictors of MASLD through a literature review and assessed these parameters in the recruited individuals. All clinical and laboratory data were measured using standard techniques and recorded in the electronic database. Medical technicians who measured the laboratory data were blinded to the results of the transient elastography. Patient data were collected during recruitment through a case record form and a review of electronic medical records. In cases where data were missing, a multiple imputation method was used to increase the statistical power and allow for the development of a valid prediction model [12].

MASLD was characterized using either a controlled attenuation parameter (CAP) value > 275 dB/m and/or a liver stiffness measurement ≥ 7.0 kPa [11,13,14,15,16]. Advanced fibrosis and cirrhosis were defined as liver stiffness measurements ≥ 8 and ≥ 10.3 kPa, respectively. The selected CAP threshold of 275 dB/m was used in this analysis due to its high efficacy in detecting steatosis among high-risk individuals without secondary causes [11]. Atherosclerotic cardiovascular disease encompassed acute coronary syndrome [17], a history of myocardial infarction, stable or unstable angina, coronary or other arterial revascularization, stroke, transient ischemic attack, or peripheral artery disease of atherosclerotic origin. Metabolic syndrome was defined as the presence of prediabetes and at least two of the following: (1) a body mass index (BMI) ≥ 23 kg/m^2^, as per the Asian-specific BMI cutoff [18,19]; (2) documented hypertension; (3) a low, sex-specific, high-density lipoprotein cholesterol (HDL-c) level or documented dyslipidemia or statin use; and (4) hypertriglyceridemia (fasting triglycerides [TG] ≥ 150 mg/dL) or documented dyslipidemia or statin use [20,21].

### 2.2. Outcomes

The primary outcome was to ascertain the prevalence of MASLD detected through transient elastography and to develop a straightforward clinical predictive score for identifying MASLD in individuals with prediabetes at high cardiometabolic risk. Additionally, we compared the performance of our predictive score with previously published scores for predicting MASLD.

### 2.3. Statistical Analysis

#### Data Collection, Imputation, and Sample Size Calculations

The sample size was calculated based on the expected prevalence of MASLD in individuals with prediabetes, estimated at 59% [22]. To estimate the prevalence with 95% confidence, a minimum sample size of 372 participants was needed [23]. Regarding the sample size for developing the predictive score, ten outcome events per predictive variable were needed. The expected prevalence in our study was sufficient to develop the predictive model.

Clinical characteristics were compared between individuals with and without MASLD. Continuous variables with a normal distribution are presented as means ± standard deviations, while continuous variables with a skewed distribution are summarized as medians and interquartile ranges. Categorical variables are shown as numbers and percentages. Statistical significance between the MASLD and non-MASLD groups was assessed using unpaired *t* tests for normally distributed continuous variables, Mann–Whitney U tests for non-normally distributed variables, and chi-square tests for categorical variables.

### 2.4. Model Development

In the initial phase of model development, univariable statistical analysis was conducted to examine the association between the collected data and MASLD. Variables with a *p* value less than 0.1 were considered to have a significant difference. In cases where two or more similar parameters were found to be correlated, the one with the lowest *p* value was selected. To simplify the model, all continuous variables were categorized using clinically meaningful cutoff values commonly utilized in practice.

Forward and backward stepwise logistic regressions were then performed to select potential predictors for the prediction model. Predictors with *p* values less than 0.05 were incorporated into the model. Finally, using clinical judgment, five predictors were selected based on their practicality for use in daily practice. Weighted scores, derived from odds ratios from the logistic regression model, were assigned to all predictors.

### 2.5. Model Performance Assessment

The performance of the developed model was assessed in terms of discrimination using unpaired *t* tests. The model’s predictive accuracy was evaluated using the area under receiver operating characteristic (AuROC) curves, sensitivity, specificity, positive predictive value, negative predictive value, positive likelihood ratio, and negative likelihood ratio. These measures were accompanied by 95% confidence intervals, calculated based on the appropriate cutoff value that aimed to achieve a balance between sensitivity and specificity. All data analyses were performed using PASW Statistics, version 18 (SPSS Inc., Chicago, IL, USA).

### 2.6. Comparison of Predictive Performance

To assess the accuracy of our scoring system in predicting the presence of MASLD in individuals with prediabetes, we compared it with five previously published noninvasive scoring systems used for MASLD detection. These were the fatty liver index (FLI) [24], the lipid accumulation product (LAP) index [25], the hepatic steatosis index (HSI) [26], the MASLD in metabolic syndrome patients score (NAFLD-MS) [27], and the NAFLD ridge score [28]. Supplementary Table S1 compares all scoring systems’ AuROCs, sensitivities, specificities, positive predictive values, negative predictive values, and likelihood ratios.

## 3. Results

### 3.1. Patient Characteristics

**Figure 1:**
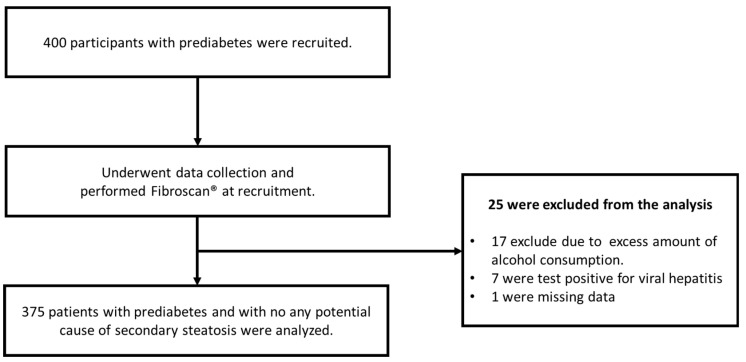
Study flow chart. Data collection and transient elastography (FibroScan Compact 530) were performed on all 400 individuals during recruitment. Twenty-five patients were excluded due to potential secondary steatosis causes and incomplete medical records.

During the recruitment period, transient elastography was performed on 400 individuals with prediabetes. Of these, 25 were excluded from the analysis due to the presence of secondary causes of hepatic steatosis. Therefore, the present study analyzed data from 375 participants (Figure 1).

Of the 375 participants, 68% were female. The overall mean age was 62.1 ± 9.9 years, and the BMI was 26.3 ± 4.6 kg/m^2^. The proportion of participants with a BMI ≥ 25 kg/m^2^ was 56.0%, and 84.0% were diagnosed with metabolic syndrome. Additionally, dyslipidemia, hypertension, and atherosclerotic cardiovascular diseases were present in 80.0%, 69.9%, and 5.3% of the participants, respectively. Regarding clinically managed metabolic disturbances, 71.1% of the participants were on statin therapy, and 88.5% used antihypertensive medications. The overall prevalence of MASLD among individuals with prediabetes at a high risk of metabolic disturbance in our study was 35.7% (*n* = 134). Moreover, the prevalence of advanced fibrosis and cirrhosis in the entire cohort were 2.4% and 1.9%, respectively. There were substantial differences in most clinical characteristics between the MASLD and non-MASLD patients. In the MASLD group, compared to the non-MASLD group, adiposity parameters (BMI, body fat percentage, fat-free mass, waist circumference, and hip circumference), the proportion of metabolic syndrome, each metabolic syndrome parameter, glycemia (FPG and HbA1C), GGT, ALT, and uric acid levels were higher. Conversely, the MASLD group had a lower HDL-c level and mean age than the non-MASLD group. The participant characteristics are outlined in Table 1.

### 3.2. Model Development

Age, adiposity parameters, FPG, HbA1c, ALT, AST, SBP, DBP, TG, and HDL-c were identified as potential predictors through univariable statistical analysis. The cutoff values of continuous data, commonly used in clinical practice to indicate clinical significance, were applied (Supplementary Table S2). Five predictors were retained in the final model following multivariable logistic regression analysis and expert consultation. They were (1) BMI ≥ 23 kg/m^2^ and BMI ≥ 25 kg/m^2^; (2) FPG ≥ 110 mg/dL; (3) HbA1C ≥ 6.0%; (4) HDL-c < 40 mg/dL in males and HDL-c < 50 mg/dL in females; and (5) ALT ≥ 30 IU/L. The results of the multivariable logistic regression, including the regression coefficients, odds ratios, and assigned scores for the final model, are detailed in Table 2. The scoring system was named the “MASLD Pre-DM score”.

### 3.3. Model Performance and Comparison of Predictive Performance of Scores in People with Prediabetes

The MASLD Pre-DM score demonstrated good discriminative power (*p* < 0.001). The parametric AuROC for the MASLD Pre-DM score was 0.80 (95% confidence interval 0.73–0.86). The sensitivity, specificity, likelihood ratio, and positive and negative predictive values at a cutoff of 4.5 are provided in Table 3.

**Figure 2:**
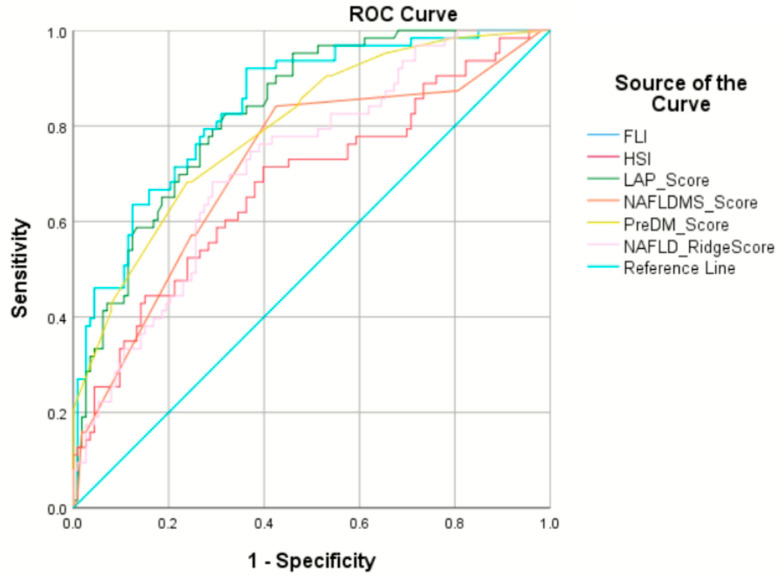
Receiver operating characteristic curves of six noninvasive scoring systems. FLI—fatty liver index (light blue line); HSI—hepatic steatosis index (red line); LAP score—lipid accumulation product index (green line); NAFLD-MS score—NAFLD in metabolic syndrome patients score (orange line); PreDM score—MASLD Pre-DM score (yellow line); NAFLD ridge score (pink line); ROC—receiver operating characteristic curve.

Comparisons of the MASLD Pre-DM score with existing scores for predicting MASLD in prediabetic individuals yielded noteworthy insights. The AuROC values for the MASLD Pre-DM, FLI, LAP index, HSI, NAFLD-MS, and NAFLD ridge scores were recorded as 0.80, 0.84, 0.83, 0.68, 0.72, and 0.72, respectively. Specifically, within the prediabetic cohort, there were no statistically significant differences in the AuROC results between the MASLD Pre-DM score and the FLI (*p* = 0.3) or between the MASLD Pre-DM and LAP index (*p* = 0.6). However, the MASLD Pre-DM’s AuROC was notably superior to those of the HSI (*p* < 0.001), NAFLD-MS (*p* = 0.005), and the NAFLD ridge score (*p* < 0.001). The performance of each noninvasive scoring system in predicting MASLD among individuals with prediabetes in our study, including the AuROC, sensitivity, specificity, likelihood ratio, and positive and negative predictive values, is detailed in Table 3 and Figure 2.

## 4. Discussion

MASLD has become the leading cause of chronic liver disease and cardiovascular disease, affecting approximately one-quarter of the population [29]. In this cross-sectional study, we engaged 375 participants at risk for cardiometabolic diseases, specifically, those with impaired fasting glucose and/or elevated HbA1c levels indicating prediabetes. Our primary objective was to determine the prevalence of MASLD using transient elastography. Additionally, we aimed to develop and evaluate a clinical predictive score for detecting MASLD in individuals with prediabetes. The overall prevalence of MASLD in our study was 35.7%. We successfully developed a simple clinical risk scoring system called the “MASLD Pre-DM score”, which effectively distinguished the presence or absence of MASLD in individuals with prediabetes. The performance of the MASLD Pre-DM score was comparable to previous scoring systems used in the general population (FLI and LAP index) and superior to a previous predictive score used in Thai participants with metabolic syndrome (NAFLD-MS).

This study is the first to investigate the prevalence of MASLD detected using transient elastography in individuals with prediabetes who are at high cardiometabolic risk. In a previous study, the prevalence of MASLD detected using ultrasonography in Indian individuals with prediabetes of average risk was 59% [22]. Our study revealed a markedly lower prevalence of MASLD at 35.7%. In further parallel with our findings, a recent extensive systematic review and meta-analysis indicated a prevalence of 32.4% for MASLD in the general adult population. Notably, the large majority (86%) of the population studied in the meta-analysis was from Asia. However, the same review found a higher prevalence of 49.2% in individuals aged 50 years or older [30].

Several factors may account for the variations in prevalence compared to our study. For instance, our study employed transient elastography, whereas most other studies relied on ultrasonography, which is known to be highly operator dependent. Furthermore, differences in participant characteristics, such as adiposity, ethnicity, metabolic parameters, and liver enzyme levels, may contribute to the variations observed. Specifically, the levels of ALT and TG in individuals with prediabetes and MASLD reported in the Indian study were higher than the corresponding levels found in our study. Our investigation’s results align with previous research highlighting the significance of ALT and TG levels as indicators of MASLD [24,25,26,27]. Such observations suggest that factors beyond prediabetes, such as ALT and TG levels, may be as pivotal in predicting MASLD as prediabetes.

Only a limited number of studies have focused on developing a predictive tool for detecting MASLD in individuals at high risk for cardiometabolic conditions and with prediabetes [31]. Since MASLD is typically asymptomatic, early detection and effective management are essential to prevent disease progression and minimize liver and cardiovascular complications. To address this need, we developed a clinical scoring system to serve as a tool for detecting MASLD in this high-risk population. Compared to other scoring systems reported in the literature, our predictive scoring system demonstrated comparable performance to the FLI and LAP index and outperformed the HIS, NAFLD-MS, and NAFLD ridge scoring systems.

Although our MASLD Pre-DM score exhibits a lower sensitivity at lower cutoff values, it offers a higher specificity than the FLI and LAP index. Although the FLI and the NAFLD-MS may provide acceptable specificity at higher cutoff values (87.1% and 99.4%, respectively), their sensitivity is very poor (48.9% and 1.2%, respectively). In resource-limited settings, allocating resources wisely and minimizing the overuse of transient elastography is crucial. An ideal scoring system should strike a balance between sensitivity and specificity. Therefore, individuals with cumulative scores of less than 4.5 can be classified into a low-probability group, where transient elastography for MASLD diagnosis may not be necessary. For individuals in the high-probability group (MASLD Pre-DM scores ≥ 4.5), the score can aid clinicians in prioritizing further MASLD investigations or making informed decisions regarding MASLD management among individuals at high cardiometabolic risk with prediabetes.

Our analysis has strengths and limitations. To our knowledge, this is the first study to investigate the prevalence of MASLD using transient elastography and to develop a practical prediction model in high cardiometabolic risk individuals with prediabetes in the Thai population. However, a limitation of our study is that we did not perform a liver biopsy, which is considered the gold standard for diagnosing MASLD. Nevertheless, previous studies have shown good comparability between transient elastography and liver biopsy for MASLD diagnosis [32,33,34,35]. Another consideration is that our scoring system was developed using data from a specific subgroup of individuals with prediabetes and high cardiometabolic risk. External validation is necessary to confirm the performance of our scoring system in more extensive and diverse subgroups, such as lean individuals, those under the age of 50, and other ethnic groups. Additionally, the potential advantages of a sex-specific scoring system, incorporating sex-related variables, merit exploration. Our findings indicated no significant sex-based differences in MASLD prevalence or most characteristics, and we incorporated sex-specific cutoffs (Supplementary Table S3). Nevertheless, the limited sex representation in our sample size necessitates further investigation with larger, more diverse cohorts.

Additionally, our scoring system focuses on detecting the presence of MASLD rather than hepatic fibrosis, which may have more substantial clinical significance for prognosis in the future. However, hepatic fibrosis is rare in individuals with prediabetes. In our study, the prevalence of advanced fibrosis and cirrhosis was low (2.4% and 1.9%, respectively). Similar results were found in another study investigating the prevalence of hepatic fibrosis in individuals with prediabetes [36]. Therefore, developing a predictive score specifically for advanced hepatic fibrosis may not be feasible. Fortunately, early detection of MASLD can help minimize the progression to hepatic fibrosis [37,38].

## 5. Conclusions

Our practical scoring system, the MASLD Pre-DM score, demonstrated its capability to predict MASLD in high cardiometabolic risk individuals with prediabetes, particularly in resource-limited settings where noninvasive transient elastography may be limited. The MASLD Pre-DM score balances sensitivity and specificity and is a simple-to-use tool for preliminary screening. With appropriate cutoff values, the MASLD Pre-DM score can benefit clinicians in the early detection of patients at risk of MASLD and increase awareness among high cardiometabolic individuals with prediabetes, potentially leading to lifestyle modifications. However, further research is needed, including external validation of the scoring system, to confirm the benefits of implementing the MASLD Pre-DM score in routine MASLD screening.

## Figures and Tables

**Table 1:** Clinical characteristics and evidence of differences (*p* values).

Characteristic	Non-MASLD	MASLD	*p*
Number	241 (64.3%)	134 (35.7%)	-
Age (years)	63.06 ± 9.43	60.46 ± 10.77	0.01
Male sex	72 (29.9%)	48 (35.8%)	0.2
BMI (kg/m^2^)	24.91 ± 3.88	28.81 ± 4.84	<0.001
Obesity * (BMI ≥ 25)	105 (43.6%)	108 (80.6%)	<0.001
Waist circumference	85.80 ± 14.16	95.08 ± 19.49	<0.001
Hip circumference	96.25 ± 10.87	103.24 ± 16.87	<0.001
Body fat percentage	31.59 ± 12.28	36.71 ± 19.27	<0.001
Fat-free mass	42.52 ± 10.81	46.41 ± 18.05	<0.001
SBP (mmHg)	130.13 ± 14.53	131.86 ± 14.01	0.2
DBP (mmHg)	71.98 ± 10.34	75.23 ± 10.94	0.005
Hypertension	148 (61.4%)	99 (73.9%)	0.01
Dyslipidemia	193 (80.1%)	108 (80.6%)	1.0
Metabolic syndrome ^†^	111 (58.4%)	102 (92.7%)	<0.001
Duration of prediabetes (years)	4 (1.0, 7.0)	4 (1.0, 7.0)	0.6
FPG (mg/dL)	94.65 ± 9.49	100.01 ± 10.85	<0.001
HbA1C (%)	5.81 ± 0.32	5.94 ± 0.35	<0.001
Triglyceride (mg/dL)	94 (71.0, 134.0)	119.5 (95.0, 156.2)	<0.001
HDL-c (mg/dL)	60.22 ± 14.90	51.03 ± 12.67	<0.001
LDL-c (mg/dL)	100.81 ± 29.56	99.31 ± 26.65	0.6
AST (IU/L)	22 (19.0, 26.0)	23 (19.0, 29.0)	0.05
ALT (IU/L)	19 (15.0, 25.0)	25 (19.0, 33.0)	<0.001
GGT (IU/L)	24 (17.0, 40.0)	34 (23.0, 59.0)	<0.001
Uric acid (mg/dL)	5.25 ± 1.07	5.73 ± 1.26	0.002
Hemoglobin	13.26 ± 1.16	13.57 ± 1.45	0.2
Platelets	252,369 ± 53,946	266,853 ± 70,524	0.2
LSM (kPa) Median (IQR)	4.6 (3.8, 5.3)	5.0 (4.37, 6.1)	<0.001
Advanced fibrosis ^‡^	0 (0%)	9 (6.7%)	<0.001
Cirrhosis ^§^	0 (0%)	7 (5.2%)	0.001
Drug–Statin	177 (73.4%)	92 (68.7%)	0.3
Number of physical activities: Moderate- to high-intensity exercise (minutes per week)	75 (0.0, 210.0)	60 (0.0, 210.0)	0.9

Data are presented as mean ± standard deviation, *n* (%), or median (25th–75th percentile). Categorical variables are shown as percentage (number), continuous normally distributed variables as mean ± standard deviation, and continuous variables with skewed distributions as median and interquartile range. ALT—alanine aminotransferase; AST—aspartate transaminase; BMI—body mass index; DBP—diastolic blood pressure; GGT—gamma-glutamyl transferase; FPG—fasting plasma glucose; HbA1c—Hemoglobin A1c; HDL-C—high-density lipoprotein cholesterol; IQR—interquartile range; IU/L—international unit per liter; kg—kilogram; kg/m^2^—kilogram per square meter; kPa—kilopascal; LDL-C—low-density lipoprotein cholesterol; LSM—liver stiffness measurement; mg/dL—milligram per deciliter; MASLD—metabolic dysfunction-associated steatotic liver disease; SBP—systolic blood pressure; SD—standard deviation. * Obesity with Asian-specific cutoff was defined by BMI ≥ 25 kg/m^2^. ^†^ Metabolic syndrome was defined as participants having prediabetes and at least 2 of the following: (1) a BMI ≥ 23 kg/m^2^, per the Asian-specific BMI cutoff; (2) documented hypertension; (3) a sex-specific low high-density lipoprotein level or documented dyslipidemia or statin use; and (4) hypertriglyceridemia (fasting ≥ 150 mg/dL) or documented dyslipidemia or statin use. ^‡^ Advanced fibrosis and ^§^ cirrhosis were defined by LSM ≥ 8 and ≥10.3 kPa, respectively.

**Table 2:** Final predictors from multivariable logistic regression, regression coefficients, odds ratios, and assigned scores.

Predictors	Cut-Off Value	Coefficient	Odds Ratio	95% CI	*p*	Assigned Score
FPG	<110 mg/dL	Reference				0
	≥110 mg/dL	1.18	3.27	2.39, 4.45	<0.001	2
HbA1C	<6.0%	Reference				0
	≥6.0%	0.81	2.24	1.83, 2.74	<0.001	1.5
HDL-c	≥40 mg/dL male ≥50 mg/dL female	Reference				0
	<40 mg/dL male <50 mg/dL female	0.74	2.10	1.67, 2.65	<0.001	1.5
ALT	<30 IU/L	Reference				0
	≥30 IU/L	0.96	2.61	2.08, 3.29	<0.001	1.5
BMI category	<23 kg/m^2^	Reference				0
	23–24.99 kg/m^2^	0.45	1.57	1.09, 2.26	0.01	1
	≥25 kg/m^2^	1.81	6.11	4.52, 8.25	<0.001	4

ALT—alanine aminotransferase; BMI—body mass index; CI—confidence interval; FPG—fasting plasma glucose; HbA1c—Hemoglobin A1c; HDL-c—high-density lipoprotein cholesterol; IU/L—international unit per liter; kg—kilogram; kg/m^2^—kilogram per square meter; mg/dL—milligram per deciliter.

**Table 3:** Performance of noninvasive scoring systems in predicting MASLD among participants with prediabetes in our study.

Predictive Performance	MASLD Pre-DM (95% CI)	LAP Index (95% CI)	FLI (95% CI)	HSI (95% CI)	NAFLD-MS (95% CI)	NAFLD Ridge (95% CI)
AuROC	0.80 (0.73, 0.86)	0.83 (0.77, 0.89)	0.84 (0.78, 0.90)	0.68 (0.59, 0.76)	0.72 (0.64, 0.80)	0.72 (0.64, 0.80)
Low cutoff						
Sensitivity (%)	70.7% (62.2%, 78.2%)	99.1% (95.0%, 100%)	93.3% (86.1%, 97.5%)	96.2% (91.4%, 98.8%)	55.6% (44.1%, 66.6%)	78.8% (70.8%, 85.4%)
Specificity (%)	72.3% (66.1%, 77.9%)	14.7% (10.0%, 20.6%)	57.9% (49.2%, 66.1%)	7.9% (4.9%, 12.1%)	74.2% (66.7%, 80.8%)	36.7% (30.6%, 43.2%)
LR (+)	2.55 (2.02, 3.22)	1.16 (1.09, 1.24)	2.21 (1.81, 2.71)	1.05 (0.99, 1.10)	2.15 (1.55, 2.99)	1.24 (1.09, 1.42)
LR (−)	0.41 (0.31, 0.53)	0.06 (0.01, 0.45)	0.12 (0.05, 0.25)	0.47 (0.18, 1.24)	0.60 (0.46, 0.78)	0.58 (0.40, 0.84)
PPV (%)	58.8% (50.7%, 66.5%)	40.2% (34.3%, 46.3%)	58.7% (50.2%, 66.9%)	36.8% (31.7%, 42.1%)	52.3% (41.3%, 63.2%)	40.9% (34.8%, 47.3%)
NPV (%)	81.5% (75.6%, 86.5%)	96.6% (82.2%, 99.9%)	93.1% (85.6%, 97.4%)	79.2% (57.8%, 92.9%)	76.6% (69.1%, 83.1%)	75.7% (66.8%, 83.2%)
High cutoff						
Sensitivity (%)			48.9% (38.2%, 59.7%)	60.2% (51.3%, 68.5%)	1.2% (0.0%, 6.7%)	78.8% (70.8%, 85.4%)
Specificity (%)			87.1% (80.4%, 92.2%)	57.7% (51.2%, 64.1%)	99.4% (96.5%, 100%)	37.1% (31.0%, 43.6%)
LR (+)			3.80 (2.35, 6.15)	1.41 (1.16, 1.74)	1.96 (0.12, 30.98)	1.25 (1.10, 1.43)
LR (−)			0.59 (0.47, 0.72)	0.69 (0.55, 0.87)	0.99 (0.97, 1.02)	0.57 (0.40, 0.83)
PPV (%)			71.0% (58.1%, 81.8%)	44.2% (36.8%, 51.8%)	50% (1.3%, 98.7%)	41.1% (35.0%, 47.4%)
NPV (%)			72.6% (65.2%, 79.2%)	72.3% (65.3%, 87.5%)	66.4% (60.0%, 72.4%)	75.9% (67.0%, 83.3%)

AuROC—area under receiver operating characteristic curve; CI—confidence interval; LR (−)—negative likelihood ratio; LR (+)—positive likelihood ratio; NPV—negative predictive value; PPV—positive predictive value.

## Data Availability

The data presented in this study are available on request from the corresponding author. The data are not publicly available due to privacy and ethical reasons.

## Supplementary Materials

The following supporting information can be downloaded at: https://www.mdpi.com/article/10.3390/jcm12247617/s1, Table S1: Noninvasive scoring systems utilized for detecting MASLD. Table S2: Univariate and multivariable logistic regression analysis of risk factors associated with MASLD in individuals with prediabetes. Table S3: Clinical characteristics and evidence of differences by sex (*p* values).
