# Supplementary material for: Clinical Predictive Score for Identifying Metabolic Dysfunction-Associated Steatotic Liver Disease in Individuals with Prediabetes Using Transient Elastography
Source: J Clin Med. 2023 Dec 11;12(24):7617. doi: 10.3390/jcm12247617 (PMC10743615; doi:10.3390/jcm12247617)
Supplement: Supplementary file 1 [file jcm-12-07617-s001.zip › jcm-2727076-supplementary.pdf]

<u>Supplementary file</u> for the manuscript entitled "**Clinical Predictive Score for Identifying Metabolic Dysfunction Associated Steatotic Liver Disease in Individuals with Prediabetes Using Transient Elastography.**"

Supplementary Table S1. Noninvasive scoring systems utilized for detecting MASLD.

Scoring system	Formulas	Cutoff value	Study population and diagnostic method for fatty liver
Fatty liver index (FLI) [1]	= (e0.953 ∗ Log e (TG) + 0.139 ∗ BMI + 0.718 ∗ Log e (GGT) + 0.053 ∗ WC - 15.745)/(1 + e0.953 ∗ Log e (TG) + 0.139 ∗ BMI + 0.718 ∗ Log e (GGT) + 0.053 ∗ WC - 15.745) ∗ 100	<30 absence of NAFLD<br>≥60 presence of NAFLD	General population, Italy, Ultrasonography
Lipid accumulation product (LAP) index [2]	Male: (WC (cm.) - 65) ∗ TG (mmol/L)<br>Female: (WC (cm.)- 58) ∗ TG (mmol/L)	Male >30.5 presence of NAFLD<br>Female >23.0 presence of NAFLD	General population, Italy, Ultrasonography
Hepatic steatosis index (HSI) [3]	= 8 ∗ ALT/AST ratio + BMI (kg/m2) + T2DM (yes = 2, no = 0) + female (yes = 2, no = 0)	<30 absence of NAFLD<br>>36 presence of NAFLD	General population, Korea, Ultrasonography
NAFLD-MS score [4]	ALT ≥ 40 U/L (yes = 2, no = 0)<br>AST/ALT ratio ≥ 1 (yes = 1, no = 0)<br>BMI ≥ 25(kg/m2) (yes = 1.5, no = 0)<br>WHR (waist to hip ratio) (≥ 0.9 in male and ≥ 0.8 in Female; yes = 1, no = 0)<br>T2DM (yes = 1, no = 0)	<3 absence of NAFLD<br>≥5 presence of NAFLD	Patients with metabolic syndrome, Thailand, Ultrasonography
NAFLD ridge score [5]	=-0.614+0.007*ALT-0.214*HDL-C+0.053*TG+0.144*HbA1C+0.032*WBC +0.132*HT	< 0.24 absence of NAFLD<br>> 0.44 presence of NAFLD	General population, Hongkong, proton-magnetic resonance spectroscopy

1. Bedogni, G.; Bellentani, S.; Miglioli, L.; Masutti, F.; Passalacqua, M.; Castiglione, A.; Tiribelli, C. The Fatty Liver Index: A Simple and Accurate Predictor of Hepatic Steatosis in the General Population. *Bmc Gastroenterol* **2006**, *6*, 33, doi:10.1186/1471-230x-6-33.
2. Bedogni, G.; Kahn, H.S.; Bellentani, S.; Tiribelli, C. A Simple Index of Lipid Overaccumulation Is a Good Marker of Liver Steatosis. *Bmc Gastroenterol* **2010**, *10*, 98, doi:10.1186/1471-230x-10-98.
3. Lee, J.-H.; Kim, D.; Kim, H.J.; Lee, C.-H.; Yang, J.I.; Kim, W.; Kim, Y.J.; Yoon, J.-H.; Cho, S.-H.; Sung, M.-W.; et al. Hepatic Steatosis Index: A Simple Screening Tool Reflecting Nonalcoholic Fatty Liver Disease. *Digest Liver Dis* **2010**, *42*, 503–508, doi:10.1016/j.dld.2009.08.002.
4. Saokaew, S.; Kanchanasuwan, S.; Apisarnthanarak, P.; Charoensak, A.; Charatcharoenwitthaya, P.; Phisalprapa, P.; Chaiyakunapruk, N. Clinical Risk Scoring for Predicting Non-alcoholic Fatty Liver Disease in Metabolic Syndrome Patients (NAFLD-MS Score). *Liver Int* **2017**, *37*, 1535–1543, doi:10.1111/liv.13413.
5. Yip, T.C. -F.; Ma, A.J.; Wong, V.W. -S.; Tse, Y. -K.; Chan, H.L. -Y.; Yuen, P. -C.; Wong, G.L. -H. Laboratory Parameter-based Machine Learning Model for Excluding Non-alcoholic Fatty Liver Disease (NAFLD) in the General Population. *Aliment. Pharmacol. Ther.* **2017**, *46*, 447–456, doi:10.1111/apt.14172.

Supplementary Table S2. Univariate and multivariable logistic regression analysis of risk factors associated with MASLD in individuals with prediabetes.

Predictors	Cutoff value	Univariable				Multivariable			
		Coefficient	Odds ratio	95% CI	p	Coefficient	Odds ratio	95% CI	p
FPG	≥110 mg/dL	1.15	3.17	1.65, 6.10	0.001	1.13	3.1	1.45, 6.65	0.004
HbA1C	≥6.0%	0.90	2.46	1.60, 3.80	<0.001	0.83	2.3	1.37, 3.66	0.001
HDL-c	<40 mg/dL male <50 mg/dL female	1.00	2.73	1.64, 4.53	<0.001	0.77	2.2	1.22, 3.82	0.008
ALT	≥30 IU/L	1.21	3.35	2.02, 5.57	<0.001	0.92	2.5	1.41, 4.48	0.002
BMI category	<23 kg/m^2	Reference				Reference			
	23-24.99 kg/m^2	0.55	1.73	0.74, 4.04	0.2	0.41	1.5	0.61, 3.71	0.3
	≥25 kg/m^2	1.96	7.11	3.58, 14.13	<0.001	1.76	5.8	2.76, 12.28	<0.001
Hypertension	Yes	0.58	1.78	1.12, 2.83	0.01	0.25	1.3	0.74, 2.22	0.3
Sex	Female	-0.27	0.76	0.49, 1.20	0.2	-0.15	0.9	0.50, 1.48	0.5

BMI, body mass index; FPG, fasting plasma glucose; HbA1c, Hemoglobin A1c; HDL-C, high-density lipoprotein cholesterol; IU/L international unit per liter; kg, kilogram; kg/m2, kilogram per square meter; mg/dL, milligram per deciliter.

Supplementary Table S3. Clinical characteristics and evidence of differences by sex (p values).

Characteristic	Male	Female	p
Number (%)	120 (32%)	255 (68%)	-
Age (years)	61.54±12.21	62.4±8.79	0.4
MASLD (%)	48 (40%)	86 (33.7%)	0.2
BMI (kg/m^2)	26.06±3.99	26.43±4.93	0.4
Obesity* (BMI ≥ 25)	67 (55.8%)	146 (57.3%)	0.7
Waist circumference	92.79±11.81	87.39±11.96	0.001
Hip circumference	98.15±8.74	99.04±10.20	0.3
SBP (mmHg)	131.99±14.86	130.16±14.12	0.2
DBP (mmHg)	76.38±9.66	71.62±10.79	<0.001
Hypertension	92 (76.6%)	155 (60.7%)	0.003
Dyslipidemia	95 (79.1%)	206 (80.7%)	0.7
Metabolic syndrome†	59 (67%)	154 (72.6%)	0.3
Duration of prediabetes (years)	2 (1.0, 7.0)	4 (1.0, 7.0)	0.4
FPG (mg/dL)	98.01±9.67	95.89±10.56	0.6
HbA1C (%)	5.82±0.37	5.87±0.32	0.1
Triglyceride (mg/dL)	105 (79, 148)	105 (78, 143)	0.6
HDL-c (mg/dL)	50.63±13.30	60.14±14.09	<0.001
Number HDL-c sex-specific cutoff (%) <40 mg/dL male, <50 mg/dL female	23 (19.1%)	56 (22.0%)	0.5
LDL-c (mg/dL)	92.63±24.03	103.89±29.80	<0.001
AST (IU/L)	24 (20, 29)	22 (18, 26)	0.004
ALT (IU/L)	24 (17, 49)	20 (16, 25)	<0.001
Drug – Statin	88 (73.3%)	181 (67.2%)	0.6

Data are presented as mean ± standard deviation, n (%), or median (25th–75th percentile). Categorical variables are shown as percentage (number), continuous normally distributed variables as mean ± standard deviation, and continuous variables with skewed distributions as median and interquartile range. FPG, fasting plasma glucose; HbA1c, Hemoglobin A1c; SBP, systolic blood pressure; DBP, diastolic BP; ALT, alanine aminotransferase; AST, aspartate transaminase; BMI, body mass index; HDL-C, high-density lipoprotein cholesterol; IQR, interquartile range; IU/L, international unit per liter; kg, kilogram; kg/m2, kilogram per square meter; kPa, kilopascal; LDL-C, low-density lipoprotein cholesterol; LSM, liver stiffness measurement; mg/dL, milligram per deciliter; NAFLD, nonalcoholic fatty liver disease; SD, standard deviation. *Obesity with Asian-specific cutoff was defined by BMI ⩾25 kg/m2. †Metabolic syndrome was defined as participants having prediabetes and at least 2 of the following: (1) BMI ⩾23 kg/m2, per the Asian-specific BMI cutoff; (2) documented hypertension; (3) a sex-specific low high-density lipoprotein level or documented dyslipidemia or statin use; and (4) hypertriglyceridemia (fasting ⩾150 mg/dL) or documented dyslipidemia or statin use.
